# Tuberculose de l’épaule masquée par une infection concomitante à enterobacter cloacae: à propos d'un cas

**DOI:** 10.11604/pamj.2015.21.9.5919

**Published:** 2015-05-05

**Authors:** Mariam Gbané-Koné, Samba Koné, Boubacar Ouali, Kouassi Jean-Mermoz Djaha, Mohamed Diomandé, Edmond Eti, Stanislas André Touré, N'zué Marcel Kouakou

**Affiliations:** 1 Service de Rhumatologie CHU de Cocody, 11 BP V13 Abidjan, Cote d'Ivoire; 2 Service Traumato-Orthopédie, CHU Cocody, 11 BP V13 Abidjan, Cote d'Ivoire

**Keywords:** Tuberculose ostéoarticulaire, épaule, Enterobacter Cloacae, osteoarticular tuberculosis, shoulder, Enterobacter Cloacae

## Abstract

La tuberculose de l’épaule est une localisation rare de même que l'arthrite septique à Enterobacter cloacae, les auteurs rapportent un cas d'ostéoarthrite de l’épaule à Bacille de Koch et à E. Cloacae chez une patiente de 36 ans avec un terrain particulier (drépanocytose SC et infection à VIH). Le diagnostic a été possible grâce aux prélèvements chirurgicaux effectués lors de l'arthrotomie

## Introduction

La tuberculose de l’épaule est rare [1]. De plus, les signes cliniques et radiologiques variables et peuvent mimer d'autres pathologies comme l'ostéomyélite chronique à pyogène ce qui pose un problème diagnostique majeur [1]. Enterobacter Cloacae (EC) est un bacille gram négatif à tropisme digestif, il est responsable d'infection nosocomiale surtout, urinaire, respiratoire, et de septicémie [2, 3]. Des rares cas d'arthrite à EC ont été rapportés dans la littérature [4]. Des arthrites tuberculeuses masquées par un germe banal sont rares, elles ont été rapportées sous forme de cas cliniques [5, 6]. Nous rapportons un cas d'ostéoarthrite tuberculeuse de l’épaule masquée par une infection à Enterobacter Cloacae. La biopsie osseuse a permis de faire le diagnostic.

## Patient et observation

Une patiente de 36 ans a été hospitalisée pour une monoarthrite chronique de l’épaule droite évoluant depuis 4 mois. Ce tableau évoluait dans un contexte de fièvre à 39- 40°, d'asthénie et d'amaigrissement non chiffré. Elle aurait reçu plusieurs traitements (antipaludiques, AINS, antibiotiques: Amoxicilline + Acide Clavulanique, Levofloxacine, Gentamycine dont la durée et la posologie n'ont pu être précisées par la patiente). Ce traitement n'a pas amélioré la symptomatologie. Cette patiente était drépanocytaire SC, et séropositive au VIH 1. Il n'y avait de porte d'entrée patente ni de notion de contage tuberculeux.

A l'examen physique, on notait une température à 38,8°, une impotence fonctionnelle absolue de l’épaule droite avec une amyotrophie des muscles de l’épaule. Aucun mouvement de l’épaule n’était possible. Localement il n'y avait pas de signes inflammatoires, ni d'adénopathies satellites. Ailleurs l'examen était sans particularité. La biologie était peu perturbée (glycémie, bilan rénal et hépatique normaux) en dehors d'une anémie hypochrome microcytaire à 09,7gr/dl. Il n'y avait pas de syndrome inflammatoire (une VS à 7 mm à H1, la CRP était négative (00mg/l). L'IDR à la tuberculine était anergique.

**Figure 1:**
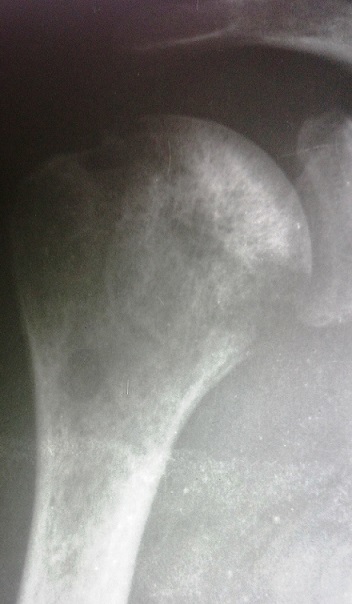
Radiographie de l’épaule droite de face aspect hétérogène de la tête humérale, avec des géodes

**Figure 2:**
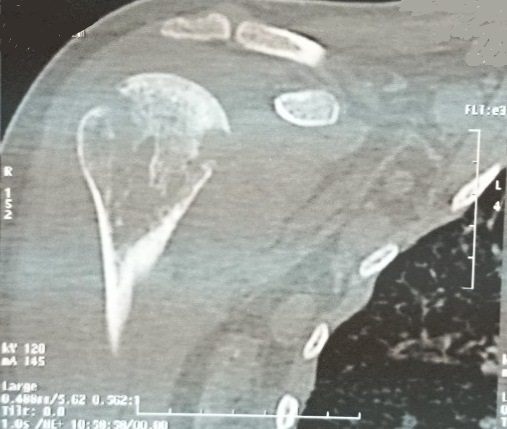
TDM épaule: érosions et géodes, séquestres de la tête humérale

A la radiographie standard des épaules, on notait un aspect hétérogène de la tête humérale droite avec une déminéralisation diffuse, et des géodes (Figure 1). Une ponction écho guidée de l’épaule avait retiré du pus franc, et l'examen bactériologique direct a isolé l'Enterobacter Cloacae sensible seulement à (l'Imipenème, Latomoxef, l'Amikacine, la Péfloxacine et la Ciprofloxacine). La patiente a été mise sous une bi antibiothérapie adaptée (imipenème+ amikacine pendant 14 jours, voie parentérale). Devant la persistance de la fièvre, une TDM de l’épaule à été réalisée, elle a mis en évidence une ostéoarthrite de l’épaule (des érosions et des géodes au niveau de la tête humérale, associées à une collection d'allure abcédée des parties molles et intra articulaires) (Figure 2).

**Figure 3:**
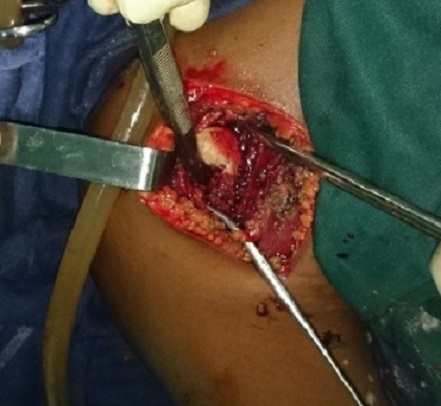
Aspect per-opératoire, nécrose de la tête humérale, abrasion du cartilage

**Figure 4:**
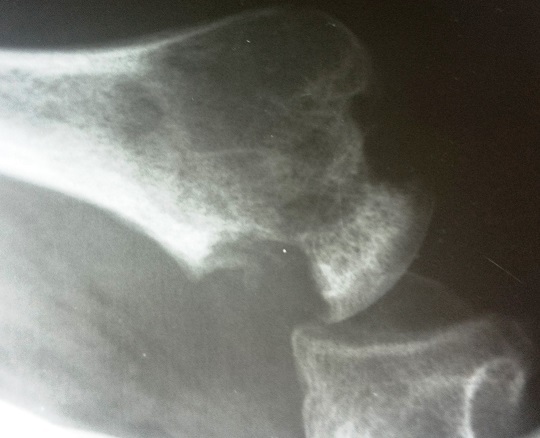
Aspect de la tête humérale à 09 mois de traitement antituberculeux

Une arthrotomie à été réalisée par le chirurgien, avec drainage des abcès, curetage biopsique et lavage articulaire (Figure 3). L'examen bactériologique direct des prélèvements chirurgicaux a retrouvé le même germe (EC). L'examen histologique quant à lui, a mis en évidence, une réaction granulomateuse faite de cellules épithélioides, de cellules géantes de Langhans et de lymphocytes, avec des plages de foyers de nécrose caséeuse. Le diagnostic final était celui d'une ostéoarthrite de l’épaule à BK et à Enterobacter Cloacae chez une patiente drépanocytaire et immunodéprimée. La patiente a été mise sous traitement antituberculeux pendant une durée d’ un an. La rééducation de l’épaule à été prescrite en post-opératoire. L’évolution immédiate a été marquée par une apyrexie franche à partir de 48 heures avec une régression progressive de la douleur. A 09 mois du traitement, elle ne présentait plus de douleur, à la radiographie on notait une stabilisation des lésions radiologiques avec des images séquellaires (Figure 4). Au plan fonctionnel, elle présentait une raideur de l’épaule avec une impotence fonctionnelle de tout le membre avec amyotrophie. Le score de constant était mauvais.

## Discussion

L'atteinte de l’épaule représente 1 à 10,5% des formes osseuses de tuberculose, elle est rare [1]. L'atteinte articulaire à *Mycobacterium tuberculosis* peut se faire soit par voie directe hématogène avec un envahissement de la membrane synoviale, soit par voie indirecte par extension d'un foyer osseux adjacent [1, 7]. L'atteinte osseuse primitive peut être difficile à diagnostiquer dans les phases précoces, augmentant le délai diagnostique [1]. Le diagnostic de tuberculose devrait être confirmé par l'isolement de *M. tuberculosis* soit lors de l'analyse histologique, soit par les cultures bactériologiques ou idéalement par les deux [1, 7].

De rares cas d'arthrite à EC ont été rapportés dans la littérature [4] avec peu d études de grande série. Une seule série de 25 cas d'infections ostéoarticulaires dues aux entérobactéries a été rapportée par Lozniewski [8] et EC y était responsable dans 22 cas. E cloacae peut être responsable d'arthrites septiques sévères, d'ostéomyélite et même de spondylodiscites [4, 8, 9]. Un cas d'ostéite calcanéenne à EC a même été rapportée chez un patient ayant une polyarthrite rhumatoïde [10].

Quelques rares cas d'arthrites tuberculeuses masquées par une infection à pyogène ont été rapportées [5, 6]. Sinnot [6] avait décrit 4 cas d'ostéomyélites tuberculeuses masquées par une infection à staphylocoque de même que Dhawan [5] avait rapporté un cas de coxite tuberculeuse masquée également par un staphylocoque. Dans tous ces cas, la présentation clinique et radiologique était celle d'une ostéoarthrite bactérienne mais comme chez notre patiente, la fièvre persistait malgré une antibiothérapie adaptée au staphylocoque.

Soulignons le rôle clé qu'a joué la chirurgie dans ce diagnostic. En effet alors que le diagnostic d'ostéoarthrite à germe banal (EC) a été confirmé avec antibiothérapie adaptée, on notait une mauvaise réponse clinique. Les prélèvements biopsiques ont permis de redresser le diagnostic en confirmant non seulement le germe banal, mais aussi une infection tuberculeuse sous jacente.

A notre connaissance notre observation est le 1^er^ cas d'une ostéoarthrite tuberculeuse masquée par E Cloacae. L'infection VIH et la drépanocytose sont probablement les facteurs favorisants de cette co-infection.

## Conclusion

L'ostéoarthrite tuberculeuse peut être masquée par une infection à germe banal. Cette co-infection peut entrainer des dégâts anatomiques importants sources de séquelles fonctionnelles invalidantes. C'est dire l'importance d'un diagnostic précoce grâce aux prélèvements chirurgicaux pour élucider ces cas cliniques inhabituels.

## References

[1] Kapukaya A, Subasi M, Bukte Y, Gur A, Tuzuner T, Kilnc N (2006). Tuberculose de l’épaule. Revue du Rhumatisme..

[2] Beaudreuil S, Hebibi H, Charpentier B, Durrbachr A (2008). Les infections graves chez les patients en dialyse péritonéale et en hémodialyse chronique conventionnelle: péritonites et infections de la voie d'abord vasculaire. Réanimation..

[3] Hiltya M, Sendia P, Seifferta SN, Droza S, Perretenc V (2013). Characterisation and clinical features of Enterobacter cloacae bloodstream infections occurring at a tertiary care university hospital in Switzerland: is cefepime adequate therapy?. Int J Antimicrob Agents..

[4] Sanders WE, Sanders CC (1997). Enterobacter spp: Pathogens Poised To Flourishat the Turn of the Century. Clin Microbiol Rev..

[5] Dhawan SS, Finks AL, Wang BWE (2009). Coxite mycobactérienne masquée par une infection concomitante à staphylocoque après un traitement par infliximab pour une maladie de Crohn. Revue du Rhumatisme..

[6] Sinnott JT, Cancio MR, Frankle MA (1990). Tuberculous osteomyelitis masked by concomitant staphylococcal infection. Arch Intern Med..

[7] Pertuiset E (2006). Tuberculose ostéoarticulaire extravertébrale. Rev Rhum..

[8] Lozniewski A, Simeon D, Lion C, Conroy MC, Mory F, Canton P, Weber M (1997). Infections ostéo-articulaires à Enterobacter spp au CHU de Nancy (1990-1994). Médecine et Maladies Infectieuses..

[9] Chassagne P, Mejjad O, Daragon A, Lecomte R, Le Loet X, Deshayes P (1990). Spondylodiscitis due to Enterobacter cloacae treated with cefixime. Presse Med..

[10] Alcaraz P, Aubran C, Jaoua C, Roudier C, Mattei JP, Announ N (2006). Ostéite septique calcanéenne par fistulisation d'un nodule rhumatoïde ulcéré. Revue du Rhumatisme..

